# Primary Adrenal Leiomyosarcoma in an Arab Male: A Rare Case Report with Immunohistochemistry Study

**DOI:** 10.1155/2015/702541

**Published:** 2015-01-19

**Authors:** Veena Nagaraj, Mohammed Mustafa, Essa Amin, Waleed Ali, Shamil Naji Sarsam, Abdulla Darwish

**Affiliations:** ^1^Department of Pathology, Bahrain Defence Force Hospital, P.O. Box 28743, Riffa, Bahrain; ^2^Department of Urology, Ibn Al Nafees Hospital, Road No. 3302, Manama, Bahrain; ^3^Department of Imaging, Ibn Al Nafees Hospital, Road No. 3302, Manama, Bahrain

## Abstract

Primary adrenal leiomyosarcoma is a rare form of adrenal mesenchymal tumors. Immunohistochemistry (IHC) together with histology takes a major role in determining the tumor type and predicting their biological behavior and differentiating them from adrenal cortical carcinoma. Appropriate radiological investigation is necessary to rule out metastatic disease from primary tumors elsewhere in the body. In this case, we report a primary leiomyosarcoma of the adrenal gland in a 61-year-old Bahraini male clinically presumed to be a renal neoplasm.

## 1. Introduction

Primary adrenal leiomyosarcomas are rare and represent 0.1-0.2% of intra-abdominal soft tissue malignancies in adults. It usually presents itself as a retroperitoneal mass with pain, mass effect, and pressure effect on the surrounding organs. Most of the adrenal mesenchymal tumors belong to benign categories like myelolipomas and haemangiomas [1]. Among malignancies, leiomyosarcoma is considered to be the most common of all tumors (25–30%). Other forms include malignant peripheral nerve sheath tumor (MPNST) and angiosarcoma [2, 3]. Diagnosis of such tumors is based on histological and immunohistological evaluation which determines the tumor type and biological behavior. In addition to the above, a complete workup of clinical, radiological, and biochemical investigations should be done to confirm the diagnosis. A microscopic examination with the aid of immunohistochemistry study is necessary for diagnosis of primary adrenal leiomyosarcoma and other tumors such as MPNST, malignant melanoma, gastrointestinal tumors (GIST), and metastatic tumors. Primary native adrenal tumors should be considered under differential diagnosis [2].

Available information on the Internet indicates approximately 20 reported cases of leiomyosarcoma around the world so far. The case of adrenal leiomyosarcoma reported in Bahrain was originally thought to be a renal tumor. However further radiological and immunohistological study led us to correct diagnosis.

## 2. Clinical History

The 61-year-old Bahraini male patient with a known history of diabetes and hypertension presented with left flank pain of few months duration. He had no other significant complaints or relevant past history. He went initially to a tertiary hospital where he was clinically diagnosed to have a renal tumor. The CT abdomen with contrast showed a large mass in the upper pole of the left kidney with heterogeneous enhancement measuring 16 × 10.7 × 11.7 cm displacing the lower pole of the kidney with mild stranding of the perinephric fat giving a radiological diagnosis of renal neoplasm.

**Figure 1:**
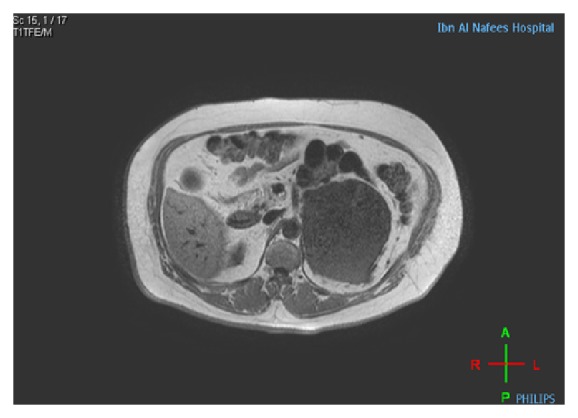
In phase axial image: 11 × 12 × 13 cm well defined hypointense lesion anterior and superior to Lt. kidney abutting the renal vessels and Lt. crus of diaphragm with no signal voids indicating calcification and no hyper intense signal indicating fat content or hemorrhage.

**Figure 2:**
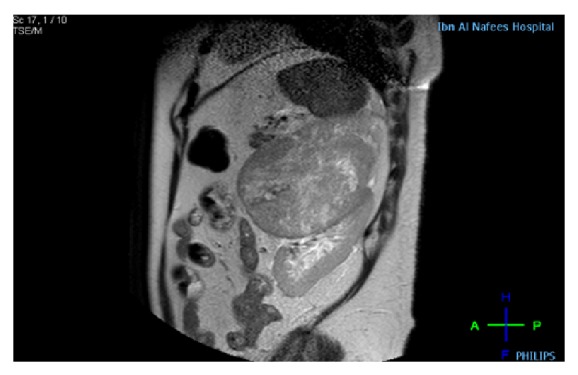
Sagittal T2W image: the lesion shows intermediate signal with multiple small hyper intense areas due to necrosis.

However, the patient went to another hospital for further investigations and treatment. Multiplanar MRI in that hospital using T1 and T2 weighted TSE SPIR postcontrast sequence showed an 11 × 12 × 13 cm oval mass with heterogeneous enhancement with nonenhancing area due to cystic degeneration in the anteromedial aspect of the left kidney displacing it posteroinferiorly. The tumor was seen abutting the splenic vein and abdominal aorta and stretching the left renal vein and artery leading to the diagnosis of adrenal tumor rather than renal origin (Figures 1 and 2). The right kidney, liver, right adrenal gland, and the gall bladder were within normal limits with no enlarged abdominal lymph nodes. Radiological differentials of pheochromocytoma, adrenal cortical carcinoma, and leiomyosarcoma were given.

The complete blood count showed mild normochromic normocytic anemia with Hb of 10.2 × 10^9^/L. Urea and electrolytes, PSA, and thyroid function test were within normal limits. The serology including autoimmune markers was negative. The midstream urine (MSU) showed RBC 3–5/HPF, WBC 2–4/HPF, and no protein, cast, crystals, or bacteria were found. Other laboratory investigations were within normal limits and no tumor marker estimations were done.

Prior to operation, according to the hospital protocol, the patient received two units of PRBCs, and then he underwent laparotomy with removal of the adrenal tumor. Intraoperatively, the tumor was arising from the left adrenal gland pushing the kidney inferiorly with no fixation to kidney or other adjacent organs. Excision of the tumor with preservation of the left kidney was performed. The patient withstood the operation well and was discharged on 5th postoperative day without any complication. Postoperatively, we lost contact with the patient as he sought further overseas oncology management.

**Figure 3:**
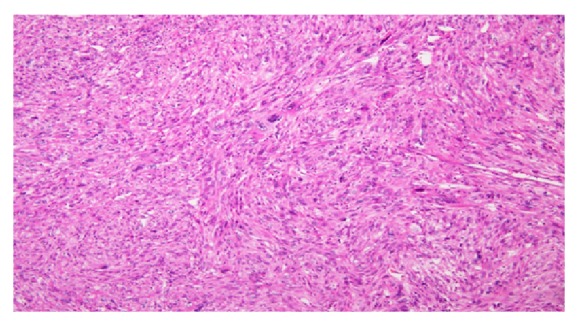
Spindly tumor cells with eosinophilic cytoplasm, blunt ended nuclei with bizarre nucleus. H&E ×40.

**Figure 4:**
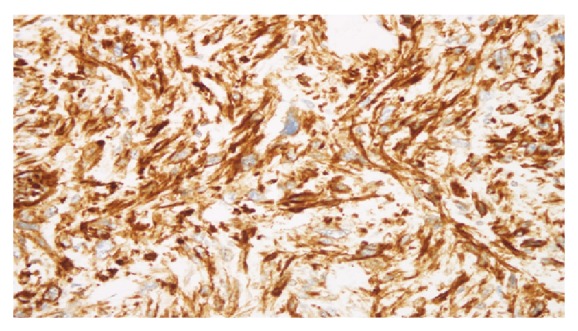
The tumor cells are strongly positive for desmin immunohistochemistry. Desmin 40x.

Grossly, multiple pieces of greyish white friable tissue with attached small amount of fatty tissue were received in the lab altogether weighing 1040 g and measuring 17 × 13 × 8 cm in maximum dimension. The cut surface was solid and grayish white in color with few mucoidal areas. Tiny hemorrhagic areas were present. No normal or residual adrenal gland was identified. Microscopic examination revealed a high grade spindle cell malignant tumor composed of diffuse proliferation of large pleomorphic spindly cells with eosinophilic cytoplasm and spindly, blunt ended vesicular nuclei (Figure 3). Many large pleomorphic cells, few multinucleate giant cells, and brisk bizarre mitosis with widespread apoptosis were present. Large areas of necrosis were noted. Considering the location of the tumor, primary adrenal cortical and medullary tumors and metastatic tumors as well as retroperitoneal tumors were considered in the differential diagnosis. Immunohistochemistry was performed which revealed that the tumor cells were strongly positive for desmin (Figure 4) and vimentin and negative for calretinin, inhibin, chromogranin, synaptophysin, S100, pan keratin, and CD68. No normal adrenal gland was present. The available adipose tissue showed no signs of tumor. Based on microscopic and immunohistochemical findings, together with the imaging studies and the confinement of the tumor to the upper pole of the kidney with no involvement of other intra-abdominal structures, a final diagnosis of primary adrenal leiomyosarcoma was given.

## 3. Discussion

Primary adrenal mesenchymal tumors are very rare and are mostly composed of benign tumors like myelolipomas and haemangiomas [1]. The most common malignant primary adrenal mesenchymal tumor is leiomyosarcoma which usually has asymptomatic presentation. Till now, only around 20 cases have been reported around the world [2–4].

The age of patients ranged from 30 to 73 years with male to female ratio of 3 : 1 and median size of the tumor is 11–25 cm [2, 4, 5]. With the cut-off size 3 cm for benign tumors, for all larger tumors, preoperative imaging and screening should be performed to assess the resectability of the tumor and the possibility of metastasis from clinically occult tumor [1]. In our case, although initially thought to be a renal tumor, further radiological study confirmed the adrenal origin of the tumor and did not reveal any tumor in other sites.

The origin of the tumor cells is widely presumed to arise from the smooth muscle wall of the inferior vena cava, central adrenal vein, and its tributaries [1, 2, 5–8]. Although the etiologies have not been clearly elucidated, association with HIV and EBV is suggested [2–6, 9, 10]. Scattered case reports suggest severe tissue trauma as the casual or contributing etiopathology in some cases. van Etten et al. suggested that a gunshot wound could be the contributing pathogenetic factor [1].

Morphological changes and clinical behaviors can be related to chromosomal aberrations [3, 6]. For example, abnormalities like 13q14-q21 loss, 5p14-pter gain, RB-1 genes, and Rb-cyclinD are some of the frequent genetic abnormalities that are associated with the shorter survival [3].

Although the tumor is unilateral, it affects both the sides equally. The patients usually present with abdominal flank pain, mass or symptoms of adjacent organ involvement like IVC obstruction leading to lower limb swelling, venous gangrene, and pulmonary embolism. They can also present with symptoms of metastasis to liver, lung, and bone leading to shortness of breath, jaundice, bone pain, and paresis [4–6, 11].

As there are no definitive biomarkers, preoperative diagnosis is difficult [4]. Although radiological features cannot differentiate among the different types of adrenal malignancies, it can be helpful in differentiating adrenal adenomas and nonadenomas by considering the size cutoff, growth rate, and characteristic radiological imagining [1, 4, 12]. Histopathology with immunohistochemistry is mandatory to determine the tumor type along with its grading and aggressiveness [3–5].

Metastatic tumors, malignant melanoma, GIST, MPNST, sarcomatoid renal cell carcinoma, malignant fibrous histiocytoma, and primary retroperitoneal sarcoma infiltrating the adrenal gland should be considered in the differential diagnosis along with the native adrenal tumors [2, 5]. Benign adrenocortical tumors are generally small and well encapsulated, weigh less than 50 gms, and recapitulate the appearance of the zona fasciculata or zona glomerulosa or both. The myelolipomas have myeloid components and mature fat and hemangiomas will show proliferating vascular components [13, 14]. Immunohistochemically benign and malignant adrenocortical carcinoma are positive for synaptophysin, inhibin, Melan-A, and calretinin [13, 14]. Malignant melanoma, GIST, MPNST, sarcomatoid renal cell carcinoma, and malignant fibrous histiocytoma will be positive for vHMB-45, c-kit, S-100, RCC marker, and CD68, respectively, and will be negative for desmin, synaptophysin, inhibin, Melan-A, and calretinin. Bilaterality is the rule when the tumor is metastatic [2, 5, 13]. Pheochromocytomas usually have cell nests with abundant basophilic to amphophilic cytoplasm and are positive for synaptophysin and chromogranin [14]. In our case, the tumor showed positivity for desmin and negativity for all the other IHC markers like synaptophysin, chromogranin, S100, calretinin, Inhibin, and pan keratin. Unilaterality of the tumor and complete replacement of the adrenal gland by the tumor were the points supporting its primary nature.

Grossly, leiomyosarcomas usually attain large size with rubbery consistency and soft if necrotic and usually show areas of hemorrhage and cystic degeneration [3–5]. Microscopically, the tumor is composed of well to poorly differentiated spindle cells, abundant pink to red cytoplasm, and centrally located blunt ended or cigar shaped nuclei [2–4]. Immunohistochemically, they show positive reaction for SMA, vimentin, desmin, calponin, and smooth muscle myosin heavy chains. They also reflect negative reaction for S100, alfa-inhibin, and CD117 [2–5].

Primary adrenal LMS can be categorized as conventional or pleomorphic type. The former being more common shows good positivity for smooth muscle markers (90–95%) while the latter pleomorphic variant shows variable expression (37.5–50%) [4, 5].

The mainstay of treatment is early diagnosis and the survival depends on tumor size, location, and complete surgical resection with free margins as well as morphologic grading. Chemotherapy and/or radiotherapy show inconsistent results [3, 4, 6]. However, van Etten et al. suggest the importance of including radiotherapy, as there are high chances of local recurrence due to the large size of the tumor and the difficulty in achieving complete resection considering its retroperitoneal location [1, 3, 6].

In spite of having slow growth and late metastasis, leiomyosarcomas carry poor prognosis and unpredictable course with high incidence of local recurrence [3]. The most important prognostic factor is the ability to achieve a microscopically negative margin. Other factors include the tumor size, location, morphological grading, venous thrombosis, and distance metastasis [1–4, 6].

In conclusion, this is a rare case of primary adrenal leiomyosarcoma which was originally thought to be a renal tumor. We stress here that when analyzing large adrenal tumor, primary soft tissue sarcomas should be considered in the differential diagnosis along with the other adrenal and metastatic tumors and appropriate immunohistochemistry should be performed to reach a specific diagnosis. Radiological investigations should also be included in the workup to reach an appropriate diagnosis and to assess the prognosis. Even with complete resection, the prognosis is generally poor and appropriate long standing follow-up is required.
